# English Teaching Quality Monitoring and Multidimensional Analysis Based on the Internet of Things and Deep Learning Model

**DOI:** 10.1155/2022/9667864

**Published:** 2022-04-28

**Authors:** Juan Song

**Affiliations:** Chongqing Metropolitan College of Science and Technology, Yongchuan, Chongqing 402167, China

## Abstract

With the development of the times, English as the universal language in the world has been highly valued by the society and schools, and English skills have become a basic skill in the society. The school is actively developing, and in the process of reform and development, the monitoring of teaching quality is essential. Teaching quality is a complex and vague concept. The establishment of a teaching quality monitoring system helps to ensure the quality of personnel training and improve the level of education and teaching, and the quality of classroom teaching is the core content of education quality. Teaching quality monitoring is the management process of various measures and actions taken to ensure the continuous improvement of students' learning quality and to achieve certain quality standards by systematically supervising and controlling various factors affecting the teaching quality in the teaching process. The research results of the article show that (1) under the traditional teaching mode, the average grades of the three groups were 74, 72, 67, 62, and 62, respectively. The score of the oral test module is relatively low, the highest score is only 63 points, the overall score shows a low level, the academic achievement is hovering on the edge of passing, and the students' English learning situation is poor. Under the new classroom quality monitoring mode, the average scores of the three groups were 96, 92, 90, 86, and 84, respectively. Compared with the ordinary teaching mode, the scores of the five detection modules were greatly improved, and the average score of the listening module was improved. The teaching contents include 22 listening modules, 20 reading modules, 23 cloze modules, 24 translation modules, and 22 speaking modules. Overall, students' English learning level has been greatly improved. (2) Generally speaking, the overall reliability coefficient of the sample data with full English teaching content is mostly kept in the range of 0.70–0.95, and only a few parts show a low situation, which also shows the overall situation of the diversity of teaching content. The overall reliability coefficient of the sample data of good teaching methods shows a relatively high situation, the reliability coefficient of the improvement of learning interest can reach 0.93, the reliability coefficient of the understanding of the learning content can reach the highest 0.96, the problem analysis ability can reach the highest 0.97, and the innovation ability can reach 0.96. The improved reliability coefficient can reach up to 0.98, which shows the authenticity and validity of the experimental data. (3) The detection result of the new classroom quality monitoring teaching mode is the highest among several models, the accuracy rate can reach 96.42%, the recall rate can reach 97.21%, and the F1 value can reach 97.46%, indicating that the new classroom quality monitoring teaching mode is effective. Teaching performance is the highest. According to the ROC curve values of the four models, we can also conclude that the ROC value of the new classroom quality monitoring teaching has been maintained at 0.98 without major twists and turns. Whether it is in the test set or the training set, the detection results of the new classroom quality monitoring are still the highest, the accuracy rate can reach 94.42%, the recall rate can reach 94.78%, and the F1 value can reach 94.49%. After the training set runs, except the performance of the traditional teaching mode increases, the performance of the other 3 models decreases.

## 1. Introduction

In order to promote the development of the global economy, the communication between all walks of life in the country is inevitable. As the carrier of communication between people, the free switching and transmission of languages in different countries are particularly important. The more complex grammar problems make the English learning process more difficult. In recent years, English has made continuous progress and innovation in education in my country. The school has also changed the original teaching mode, actively improved the teaching ability of teachers, strived to cultivate students' innovative ability, and constantly developed new teaching methods and improved the quality of English teaching. In teaching, English teachers should choose appropriate teaching methods, which play a key role in improving teachers' teaching ability and improving students' English performance. According to the situation of different students, we should teach students according to their aptitude and improve the quality of teaching. Literature [1] studied the theory of deep learning and proposed a blended learning strategy to encourage students to achieve deep learning. Reference [2] revolved around deep learning model-based IICS anomaly detection techniques using information collected from TCP/IP packets for learning and validation. In Reference [3], a computer framework facilitates the inference of distributed deep learning models and is executed cooperatively by devices in a distributed computing hierarchy. Reference [4] showed that vernacular teaching reduces the value of the reflective practitioner spirit among teachers and thus hinders the initiative of CPD among university teachers. Literature [5] studied the application of data mining technology in teaching system management, discussed the current teaching quality evaluation system in colleges and universities, and proposed that the embedded system in the Internet of Things can transmit the data from the sensor layer to the network layer, so as to realize the integration between the sensor and the Internet. Quality management is the core task of teaching, and the establishment of monitoring system is an important content of quality management [6]. The quality of learning and teaching depends on many factors, the most important being learner self-assessment and formal assessment or testing, monitoring learner success and achievement, teacher development, and teacher evaluation[7]. Reference [8] proposed a deep learning-based IoT real-time health monitoring system using cross-testing to extensively evaluate the performance of the proposed system. Reference [9] introduced two different machine learning-based algorithms for photovoltaic (PV) array fault monitoring and classification. The study demonstrated the capability and feasibility of the method. Reference [10] illustrated a brand-new listing and oral course model based on the Tell Me More teaching system based on a blended English teaching model. Literature [11] studied and analyzed the influencing factors of college English classroom teaching quality evaluation and formed a relatively complete and suitable college English classroom in China. Reference [12] illustrated some potential and noteworthy issues and advantages of e-learning models applied to English teaching. Reference [13] demonstrated the feasibility of building IoT applications powered by effective, efficient, and reliable deep learning models. Reference [14] emphasized the importance of the English teaching quality monitoring system. Literature [15] proposed three aspects: teachers' professional ethics and habits, giving full play to teachers' leading role, and improving students' performance evaluation system and English teaching.

## 2. English Teaching Quality Monitoring and Analysis

### 2.1. Status Quo of English Teaching Quality

There are many factors in the process of English teaching. First of all, the purpose of English teaching must be clear, so that students can gain some perceptual knowledge of English, stimulate their interest in English learning and cultivate their ability, and encourage students to speak English boldly, so as to lay a solid foundation, to develop good study habits to lay the foundation for further study of English. In the process of English teaching, we cannot rely solely on teachers' simple explanations and students' listening methods. We should change the traditional teaching methods and have appropriate oral interaction with students. Teachers should update English teaching concepts appropriately in teaching, further study the teaching concepts in the English curriculum standards, highlight the practicality of language, and should use less mechanical memory and more flexible use in teaching. Taking advantage of the Internet, it is possible to learn English universally anytime, anywhere. An English teaching quality monitoring system based on the Internet of Things and deep learning models is established, and a scientific and standardized comprehensive teaching quality management system is built.

### 2.2. Teaching Quality Monitoring System

The teaching quality assurance system in colleges and universities is to use the concepts and methods of system theory to organize the functions of various stages and links of quality management in order to achieve the training objectives and to implement institutionalized, structured, and continuous monitoring of personnel training activities. The teaching process is evaluated and diagnosed, and a stable and effective quality management system is formed with clear tasks, responsibilities, and authorities that coordinate and promote each other to ensure and improve the quality of teaching. Specifically implemented in the implementation process of college management, it mainly includes the teaching objective system, the curriculum system of colleges and universities, the teacher system of colleges and universities, and the evaluation system. To establish a perfect teaching quality assurance system in colleges and universities, it is necessary to ensure the effective and harmonious development of these subsystems. To ensure that the objects you monitor can reach the standard in every teaching link, only when every small link in the front meets the standard and lays a good foundation, you can reap the full benefits in the final major link. In this way, under the complete set of scientific teaching quality monitoring system, the teaching process can be improved towards the established goals and its shortcomings can be improved to form high-efficiency and high-quality teaching. The evaluation indicators for constructing the teaching quality of efficient teachers are shown in Table 1.

### 2.3. The Role of the Teaching Monitoring System in Teaching

Through the cloud education and teaching quality evaluation and monitoring system, scientific analysis and diagnosis of the effects and existing deficiencies of all aspects of teaching are carried out to provide reference for teaching decision-making or improvement [21]. It reflects the teaching effect of teachers and the learning effect of students, so as to encourage them to further improve their teaching ability and learning methods. For teachers, it is difficult for teachers to find their own shortcomings in the teaching process. Teaching monitoring can solve these problems very well. It can not only enable teachers to find their own problems in time, correct them, and create new teaching methods, but also form efficient and high-quality English classroom teaching. It is also possible to continuously tap the potential of teachers, understand their own advantages, and carry them forward. For students, complete teaching quality standards can help students better complete teaching tasks, guide students in the direction of learning, make each student master their own learning methods, and formulate different learning foundations for each student. The goal of learning is to enable students to study with high quality and high efficiency.

## 3. Quality Monitoring of English Teaching Based on Internet of Things Technology

### 3.1. Establishment of Monitoring Model

The English teaching quality monitoring adopts the maximum and minimum value method in normalization, which can well preserve the original meaning of the data. The calculation formula is as follows:

(1) $$\begin{array}{c} X=\frac{I-I_{\min}}{I_{\max}-I_{\min}}. \end{array}$$

Among them, *X* is the normalized score, *I*_min_ is the minimum teaching quality score, *I*_max_ is the maximum score, and *I* is the unprocessed score.

English learning error formula is as follows [22]:

(2) $$\begin{array}{c} E=\frac{\sum_{k=1}^{\rho }\sum_{j=1}^{l}\left(y_{j}^{k}-o_{j}^{k}\right)}{2},\\ \text{fitness}=\frac{1}{E}. \end{array}$$

The formula for calculating the probability of selection is as follows [23]:

(3) $$\begin{array}{c} z\left(a_{j}\right)=\frac{f\left(a_{j}\right)}{\sum_{j=1}^{d}f\left(a_{j}\right)}. \end{array}$$

English teaching quality monitoring is as follows:

(4) $$\begin{array}{c} q\left(a_{k}\right)=\sum_{j=1}^{k}z\left(a_{j}\right). \end{array}$$

Sample of classroom teaching quality evaluation is as follows [24]:

(5) $$\begin{array}{c} E=\frac{1}{n}\sum_{j=1}^{n}\left|\sum_{k=1}^{m}{\left(B_{jk}-A_{jk}\right)}^{2}\right|. \end{array}$$

Normalized processing is as follows:

(6) $$\begin{array}{c} X^{*}\left(k\right)=\frac{x_{k}-x_{k\min}}{x_{k\max}-x_{k\min}}. \end{array}$$

The original English teaching quality evaluation data are standardized, and the calculation is published as follows:

(7) $$\begin{array}{c} x_{ij}^{\prime }=\frac{\left(x_{ij}-\bar{x}\right)}{S_{j}}. \end{array}$$

Normalized value is as follows [25]:

(8) $$\begin{array}{c} Z_{ij}=x_{ij}^{\prime }+A. \end{array}$$

The same quantification of teaching quality evaluation indicators is as follows:

(9) $$\begin{array}{c} p_{ij}=\frac{Z_{ij}}{\sum_{i=1}^{m}Z_{ij}},\;\left(i=1,2,\ldots ,m;j=1,2,\ldots ,n\right). \end{array}$$

The index entropy value *E*_*j*_ is calculated as follows:

(10) $$\begin{array}{c} E_{j}=-k\sum_{i=1}^{m}p_{ij}\ln\left(p_{ij}\right), \end{array}$$

in

(11) $$\begin{array}{c} k=\frac{1}{\ln\left(n\right)},\;E_{j}\geq 0. \end{array}$$

The coefficient of variance *G*_*j*_ is calculated as follows:

(12) $$\begin{array}{c} G_{j}=1-E_{j}. \end{array}$$

Indicator weight *w*_*j*_ is calculated as follows:

(13) $$\begin{array}{c} w_{j}=\frac{G_{j}}{\sum_{j=1}^{n}G_{j}}. \end{array}$$

The teaching quality of the sample *F*_*i*_ is calculated as follows:

(14) $$\begin{array}{c} F_{i}=\sum_{j=1}^{n}w_{j}p_{ij}. \end{array}$$

### 3.2. Establishment of English Teaching Quality Model

Calculating the normalization of English teaching quality indicators,

(15) $$\begin{array}{c} {\bar{u}}_{ij}=\frac{u_{ij}}{\sum_{k=1}^{n}u_{kj}}. \end{array}$$

The canonical average is found as follows:

(16) $$\begin{array}{c} {\hat{w}}_{i}=\frac{1}{n}\sum_{j=1}^{n}u_{ij}. \end{array}$$

English teaching evaluation index is as follows:

(17) $$\begin{array}{c} CI=\frac{\lambda_{\max}-n}{n-1}. \end{array}$$

Final evaluation index is as follows:

(18) $$\begin{array}{c} \lambda_{\max}=\frac{1}{n}\sum_{i=1}^{n}\frac{\left(U\hat{W}\right)}{{\hat{W}}_{i}}. \end{array}$$

## 4. Simulation Experiments

### 4.1. Comparative Experiment

**Figure 1:**
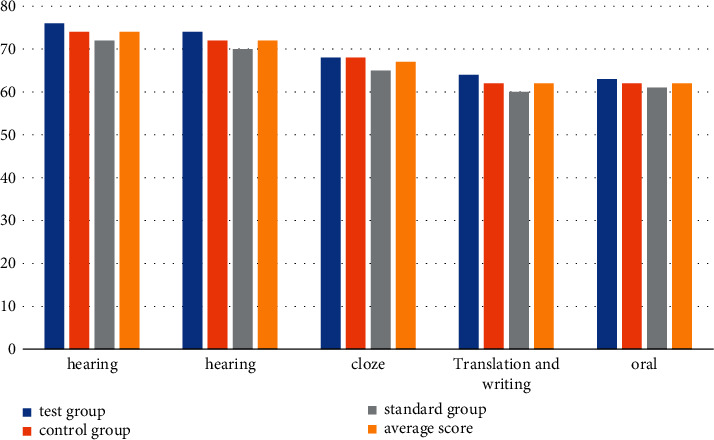
Statistics of the traditional teaching mode.

In order to make the experimental data more convincing, we selected 3 teachers with the same teaching age to teach the students in the 3 groups, respectively, and the teaching duration was one academic year. The experiment compares the student achievement of the traditional teaching mode with the student achievement of the new classroom quality monitoring mode and observes the superiority of the new classroom quality monitoring mode in English education. Before the start of the experiment, in order to ensure the objectivity of the experimental data, the three groups were tested separately, including the written part (listening, reading, gestalt, translation, and composition) and the oral part. The written part was graded by two teachers, and the oral part was taken. Generally, the average score of two teachers is taken. The specific experimental data are shown in Table 2 and Figure 1.

From the data in Table 2, we can conclude that under the traditional teaching mode, the average scores of the experimental group, the control group, and the standard group are 74, 72, 67, 62, and 62, respectively, and there is no significant difference in the scores of the three groups. Among them, the score of the experimental group is the highest among the three groups, and the listening modules of the three groups are the highest among all the detection modules. The listening score of the experimental group can reach 74 points, and the listening score of the control group is 74 points; the listening score of the standard group is 72 points. The score of the oral test module is relatively low, the highest score is only 63 points, the overall score shows a low level, the academic achievement is hovering on the edge of passing, and the students' English learning situation is poor.

**Figure 2:**
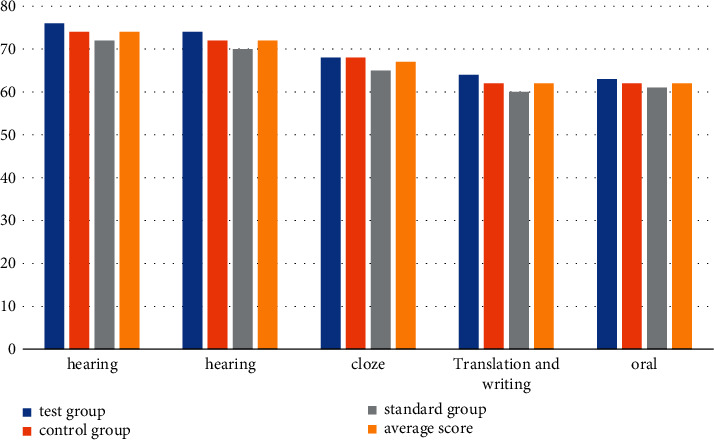
Results statistics of the new classroom quality monitoring model.

According to the data in Table 3 and Figure 2, we can conclude that under the new classroom quality monitoring mode, the average scores of the experimental group, the control group, and the standard group are 96, 92, 90, 86, and 84, respectively. The speaking module is still the highest among the testing modules. The oral score of the experimental group is 98, the score of the control group is 96, and the score of the standard group is 94. Compared with the traditional teaching model, the scores of the five testing modules are all there was a big improvement, with a 22 average improvement in listening modules, a 20 average improvement in reading modules, a 23 average improvement in cloze modules, a 24 average improvement in translation and writing modules, and a 24 average improvement in speaking modules. The score is 22. Generally speaking, the English learning level of the students has been greatly improved. The experimental results also show that the new classroom quality monitoring mode can improve the teaching quality.

### 4.2. Simulation Experiment

The experiment combines the results of teachers' English teaching evaluation to find out the factors that affect English teaching. The experiment adopts the method of questionnaire survey. According to the collected questionnaire results, the results are divided into two indicators. The first-level indicators can be subdivided into five. Grades and secondary indicators can be subdivided into 18 grades, the developed questionnaires are scored by students, and the results are collected to obtain experimental data. The experiment is scored from two aspects: teaching content and teaching method. The experimental results are as follows.

#### 4.2.1. Teaching Content

**Figure 3:**
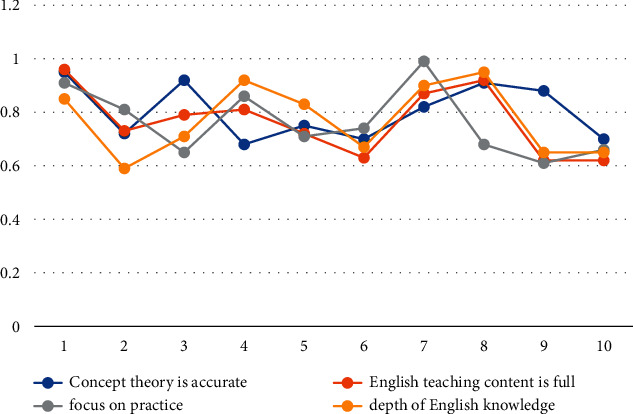
Sample results of teaching content.

According to the data in Table 4 and Figure 3, we can conclude that the conceptual theoretical accuracy reliability coefficient of sample No. 1 has reached 0.95, and the other reliability coefficients of No. 1 are also maintained above 0.85, indicating the authenticity of the experimental data. In general, the overall reliability coefficient of the sample data with full English teaching content is mostly kept in the range of 0.70–0.95, and only a few parts show a low situation, which also shows that the overall situation of the diversity of teaching content is good. The reliability coefficient of practice is generally in a high state, and the reliability coefficient of deep understanding of English knowledge can reach a maximum of 0.95. In general, the test results of teachers' teaching content are in a good state, and the diversity of teachers' teaching content is good.

#### 4.2.2. Teaching Methods

**Figure 4:**
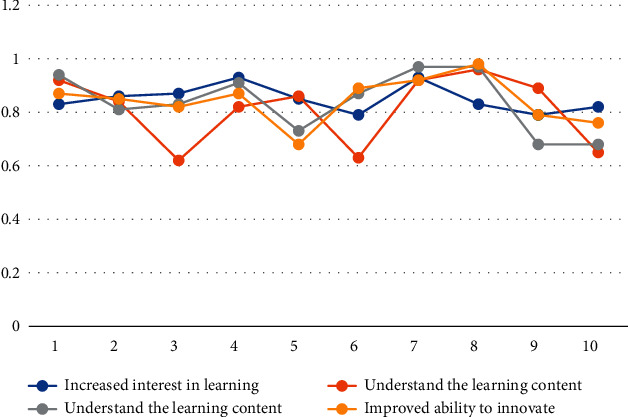
Teaching method sample results.

According to the data in Table 5 and Figure 4, we can conclude that the overall reliability coefficient of the teaching method sample data shows a high situation, the reliability coefficient of the improvement of problem analysis ability can reach 0.93, and the reliability coefficient of the understanding of the learning content is the highest. Reaching 0.96, the analysis ability can be improved up to 0.97, and the reliability coefficient of innovation ability can be up to 0.98, which shows the authenticity and validity of the experimental data. Among them, the reliability coefficient of sample data 1 shows a high state.

### 4.3. Model Performance Check

In the experiment, the model proposed in the article and other teaching models are run in different dimensions to test the superiority of the model. The experimental method is to run the four models in two test sets in turn. Among them, the training set is the sample set which is set aside during the model training process, which can be used to adjust the hyperparameters of the model and evaluate the ability of the model. However, the test data set is different. Although it is the same sample set which is set aside during the model training process, it is used to evaluate the performance of the final model, helping to compare multiple final models and make choices. The ROC curve combines the sensitivity and specificity in a graphical way, which can accurately reflect the relationship between the specificity and sensitivity of the analytical method. It is a comprehensive representative of the test accuracy and can represent the performance of each model in the article. The specific experimental data are shown in Tables 6 and 7.

**Figure 5:**
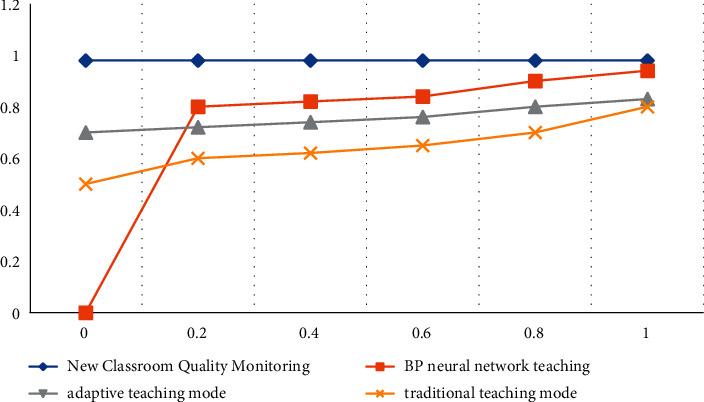
ROC curve on the test set.

According to the data in Table 6 and Figure 5, we can conclude that the detection result of the new classroom quality monitoring teaching mode proposed by the article is the highest among several models, the accuracy rate can reach 96.42%, the recall rate can reach 97.21%, and the F1 value can reach 97.46%, indicating that the teaching performance of the new classroom quality monitoring teaching mode is the highest. Among them, the experimental results of the traditional teaching mode show a lower state, which indicates that the students' English learning effect is not ideal under the traditional teaching mode. The detection values of BP neural network teaching and adaptive teaching mode remain in the middle of the two. According to the ROC curve values of the four models, we can also conclude that the ROC value of the new classroom quality monitoring teaching has been kept at 0.98 without major twists and turns. The ROC curve of the BP neural network teaching is more tortuous, and the ROC value is relatively unstable. The description is less accurate.

**Figure 6:**
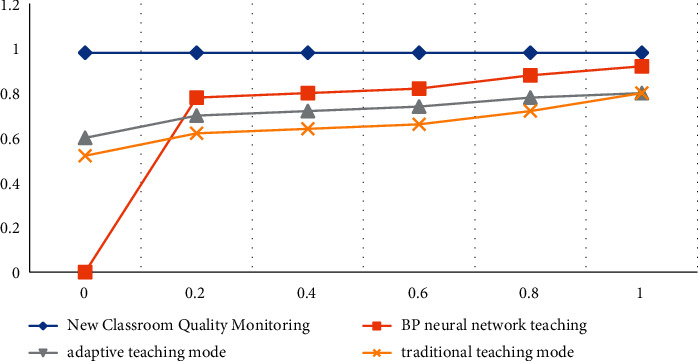
ROC curve on the training set.

According to the data in Table 7 and Figure 6, we can conclude that, whether in the test set or the training set, the detection results of the new classroom quality monitoring are still the highest, the accuracy rate can reach 94.42%, the recall rate can reach 94.78%, and the F1 value can reach 94.49%. After the training set runs, the detection effect of the model decreases to a certain extent, and the detection result of the traditional method improves to a certain extent due to the long use time. According to the ROC curves of the four models, we can also see that the ROC value of the new classroom quality monitoring has been stable at 0.98 without major twists and turns. The ROC curves of the other three models are more tortuous, and the ROC values are lower. It also shows that the recognition accuracy of the new classroom quality monitoring model is the highest.

## 5. Conclusion

English has received extensive attention from society and schools as the world's universal language. English has been regarded as a basic skill to enter the society, but some universities still have the problem of English teaching defects, because English is not our mother tongue, the particularity of English teaching, English teaching has not received enough attention, schools lack excellent teacher resources, and students cannot enjoy high-quality education. Since English involves a lot of grammar problems, there are many problems in all English teaching process. All students should gradually cultivate their English reading habits. They can listen to more English songs and watch English movies, which is convenient and better to improve the effect of classroom teaching. Although the English teaching quality monitoring model in this paper can monitor the English learning situation, it needs to be further improved in terms of teaching evaluation. We believe that the establishment of a scientific college English teaching quality monitoring system will not only improve the quality of college English teaching but also promote the rapid integration of Chinese college education with international college education and can also avoid language barriers.

## Figures and Tables

**Table 1:** Teaching quality evaluation indicators.

First-level indicator	Secondary indicators
Teaching content	The teaching objectives are clear and meet the teaching content requirements.
	Teachers have a thorough understanding of basic theories, concepts, key points, and difficult points [16].
	The content is substantial, the arrangement is reasonable, and the introduction of new achievements is emphasized [17].

Teaching skills	The layout of the blackboard writing is reasonable, the levels are clear, and the content is clear.
	The language is fluent, accurate, and clear.
	Teachers adopt a variety of teaching methods, teach students in accordance with their aptitude, and focus on cultivating students' ability to analyze and solve problems [18].

Teaching attitude	The teaching work is enthusiastic, full of energy, serious, and devoted.
	Abide by the teaching discipline, get to and from class on time.
	Teachers are rigorous in their studies, strict in teaching, and well-mannered.

Teacher's knowledge level and research ability	The knowledge is extensive, and the teaching can absorb the theory and knowledge of multiple courses.
	Have high scientific research ability.
	Have a certain scientific research ability.

Appraisal and performance	The assessment indicators are scientific and adopt a combination of diagnostic, procedural, and summative methods [19].
	Carefully correct students' experimental reports, with assessment analysis and summary.
	The classroom atmosphere is active and students are more motivated to learn.
	Students can operate experiments independently and correctly and have a solid grasp of basic knowledge [20].

**Table 2:** Results statistics of traditional teaching mode.

Group	Hearing	Hearing	Cloze	Translation and writing	Oral
Test group	76	74	68	64	63
Control group	74	72	68	62	62
Standard group	72	70	65	60	61
Average score	74	72	67	62	62

**Table 3:** New classroom quality monitoring mode.

Group	Hearing	Hearing	Cloze	Translation and writing	Oral
Test group	98	94	92	88	86
Control group	96	92	90	86	84
Standard group	94	90	88	84	82
Average score	96	92	90	86	84

**Table 4:** Sample data of teaching content.

Sample number	Concept theory is accurate	English teaching content is full	Focus on practice	Depth of English knowledge
1	0.95	0.96	0.91	0.85
2	0.72	0.73	0.81	0.59
3	0.92	0.79	0.65	0.71
4	0.68	0.81	0.86	0.92
5	0.75	0.72	0.71	0.83
6	0.70	0.63	0.74	0.67
7	0.82	0.87	0.99	0.90
8	0.91	0.92	0.68	0.95
9	0.88	0.62	0.61	0.65
10	0.70	0.62	0.66	0.65

**Table 5:** Sample data of teaching methods.

Sample number	Increased interest in learning	Understand the learning content	Understand the learning content	Improved ability to innovate
1	0.83	0.92	0.94	0.87
2	0.86	0.84	0.81	0.85
3	0.87	0.62	0.83	0.82
4	0.93	0.82	0.91	0.87
5	0.85	0.86	0.73	0.68
6	0.79	0.63	0.87	0.89
7	0.93	0.92	0.97	0.92
8	0.83	0.96	0.97	0.98
9	0.79	0.89	0.68	0.79
10	0.82	0.65	0.68	0.76

**Table 6:** Test set experimental results.

Model	Accuracy (%)	Recall (%)	F1 score (%)
New classroom quality monitoring	96.42	97.21	97.46
BP neural network teaching	92.41	93.12	93.24
Adaptive teaching mode	88.24	87.14	84.23
Traditional teaching mode	79.21	80.24	81.23

**Table 7:** Experimental results of training set.

Model	Accuracy (%)	Recall (%)	F1 score (%)
New classroom quality monitoring	94.42	94.78	94.49
BP neural network teaching	90.23	90.92	91.24
Adaptive teaching mode	85.46	85.89	86.19
Traditional teaching mode	82.46	83.10	83.78

## Data Availability

The experimental data used to support the findings of this study are available from the corresponding author upon request.
